# Effects of electric field and strain engineering on the electronic properties, band alignment and enhanced optical properties of ZnO/Janus ZrSSe heterostructures

**DOI:** 10.1039/d0ra00917b

**Published:** 2020-03-06

**Authors:** Dat D. Vo, Tuan V. Vu, Thi H. Tham Nguyen, Nguyen N. Hieu, Huynh V. Phuc, Nguyen T. T. Binh, M. Idrees, B. Amin, Chuong V. Nguyen

**Affiliations:** Division of Computational Physics, Institute for Computational Science, Ton Duc Thang University Ho Chi Minh City Vietnam voduydat@tdtu.edu.vn; Faculty of Electrical & Electronics Engineering, Ton Duc Thang University Ho Chi Minh City Vietnam; Center of Excellence for Green Energy and Environmental Nanomaterials, Nguyen Tat Thanh University Ho Chi Minh City Vietnam; Institute of Research and Development, Duy Tan University Da Nang 550000 Vietnam nguyentthanhbinh8@duytan.edu.vn; Division of Theoretical Physics, Dong Thap University Cao Lanh 870000 Vietnam; Department of Physics, Hazara University Mansehra 21300 Pakistan; Department of Physics, Abbottabad University of Science and Technology Abbottabad 22010 Pakistan; Department of Materials Science and Engineering, Le Quy Don Technical University Ha Noi 100000 Vietnam chuongnguyen11@gmail.com

## Abstract

The formation of van der Waals heterostructures (vdWHs) have recently emerged as promising structures to make a variety of novel nanoelectronic and optoelectronic devices. Here, in this work, we investigate the structural, electronic and optical features of ZnO/ZrSSe vdWHs for different stacking patterns of ZnO/SeZrS and ZnO/SZrSe by employing first-principles calculations. Binding energy and *ab initio* molecular dynamics calculations are also employed to confirm the structural and thermal stability of the ZnO/ZrSSe vdWHs for both models. We find that in both stacking models, the ZnO and ZrSSe layers are bonded *via* weak vdW forces, leading to easy exfoliation of the layers. More interestingly, both the ZnO/SeZrS and ZnO/SZrSe vdWHs posses type-II band alignment, making them promising candidates for the use of photovoltaic devices because the photogenerated electrons–holes are separated at the interface. The ZnO/ZrSSe vdWHs for both models possess high performance absorption in the visible and near-infrared regions, revealing their use for acquiring efficient photocatalysts. Moreover, the band gap values and band alignments of the ZnO/ZrSSe for both models can be adjusted by an electric field as well as vertical strains. There is a transformation from semiconductor to metal under a negative electric field and tensile vertical strain. These findings demonstrate that ZnO/ZrSSe vdWHs are a promising option for optoelectronic and nanoelectronic applications.

## Abstract 2

Here, in this work, we investigate the structural, electronic and optical features of ZnO/ZrSSe vdWHs for different stacking patterns of ZnO/SeZrS and ZnO/SZrSe by employing first-principles calculations.

## Graphical abstract

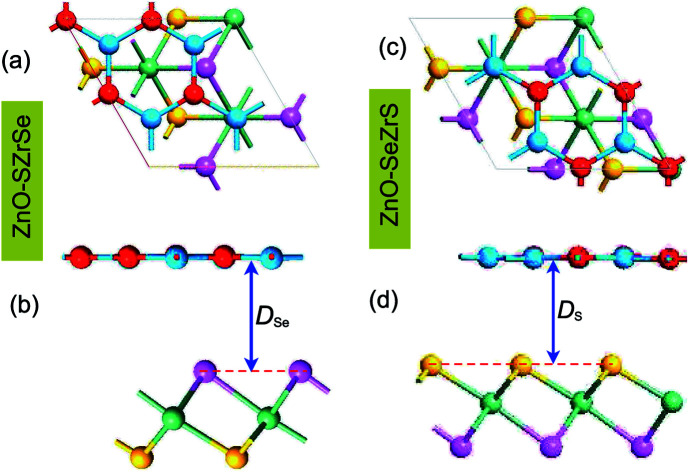

## 1 Introduction

Graphene^1^ and other graphene-like two-dimensional (2D) analogues, such as silicene,^2^ transition metal dichalcogenides (TMDs),^3,4^ and phosphorene^5,6^ have gained a lot of attention from the scientific community, not only experimental but also theoretical research, because of their outstanding electronic and optical properties which make them promising candidates for technological advances. For instance, a high carrier mobility of 2 × 10^5^ cm^2^ V^−1^ s^−1^ (ref. 7) of graphene makes it promising for high-performance electronic devices, such as field effects transistors (FETs).^8,9^ Strong in-plane anisotropy in the electronic and optical properties of phosphorene^10^ predict that it could also be a promising candidate for FETs. Unfortunately, these 2D materials have several drawbacks, which restrict their applications in electronic and nanoelectronic devices. For example, the absence of a sizable band gap in graphene is a considerable limitation for its use as a logic circuit. The low carrier mobility of TMDs monolayers (200 cm^2^ V^−1^ s^−1^) is one of the serious limitations for its use in metal-oxide semiconductor FETs.^11^

Currently, there have been many common strategies, such as strain engineering,^12–14^ electric field,^15^ stacking layers^16,17^ and so forth, that can be effectively used to reduce the limitations in the above-mentioned 2D materials. Among these, the creation of 2D van der Waals heterostructures (vdWHs) by stacking different 2D materials on top of each other is known to be one of the most efficient strategies to modulate electronic properties and to enhance the optical features of the constituent 2D materials.^18–21^ One can observe that the weak vdW interactions in such vdWHs keep them energetically stable and preserve the superior advantages of the constituent monolayers. Moreover, the 2D vdWHs also exhibit many new physical and chemical properties, which may not hold in the single materials. In recent years, experimentalists have been devoted to fabricating several vdWHs based on different 2D materials for use in high-efficiency optoelectronic and nanoelectronic devices. At the same time, theorists have been trying to construct different 2D vdWHs and explore the underlying electronic and optical features and provide significant guidance on 2D vdWHs designs for next-generation nanodevices. To date, several vdWHs based on different 2D materials have been fabricated experimentally and investigated theoretically, such as SnSe_2_/MoS_2_,^22,23^ graphene/MX (M = Ga, Ge; X = S, Se, Te),^24–28^ and TMDs/phosphorene.^29–31^ These studies demonstrates that the vdWHs based on different 2D materials exhibit many novel excellent electronic and optical features, that merit novel nanoelectronic and optoelectronic applications.

Recently, a new type of TMDs material, namely 2D Janus materials, have gained considerable interest from scientists owing to their large intrinsic dipole and strong Rashba spin–orbit coupling, which are absent in the parent TMD materials. It is interesting that Janus TMDs, such as MoSSe monolayers, have recently been synthesized by sulfurization of MoSe_2_ (ref. 32) or selenization of MoS_2_.^33^ To date, several Janus materials have been developed, such as Janus PtSSe,^34,35^ SnSSe^36^ and ZrSSe^37,38^ monolayers. Guo *et al.*,^37^*via* first-principle calculations demonstrated that the Janus ZrSSe monolayer is dynamically and mechanically stable. Vu *et al.*^38^ showed that the Janus ZrSSe monolayers exhibit photocatalytic activity, which is suitable for water splitting applications.

More recently, 2D graphene-like ZnO has received enormous interest owing to its excellent electronic, piezoelectric and optical properties, such as a large band gap value and exciton binding energy.^39^ 2D ZnO is known to be an environmentally friendly material, which has been successfully synthesized in experiments,^40–42^ making it a desirable material for fabricating high-efficiency photovoltaic and optoelectronic nanodevices. To date, vdWHs made by stacking ZnO monolayers on top of another 2D material have been proposed and widely investigated, such as ZnO/phosphorene,^43,44^ ZnO/TMDs,^45–47^ ZnO/BSe,^48^ and ZnO/GaN.^49^ These investigations demonstrated that 2D ZnO can be considered as an ideal material for constructing vdWHs. For instance, Ren *et al.*^48^ used first principles calculations to consider the electronic and optical properties of the ZnO/BSe vdWH. They demonstrated that the ZnO/BSe vdWH is a promising material for water splitting because it possesses a type-II band alignment with high carrier mobility and enhanced optical absorption. Wang *et al.*^45^ studied several kinds of ZnO/TMDs vdWHs, including ZnO/MX_2_ (M = Mo, W; X = S, Se) and demonstrated that all these vdWHs display excellent optical absorption, which merits optical and photovoltaic applications.

Therefore, in this work, we first construct ultrathin vdWH by vertically stacking ZnO on top of Janus ZrSSe monolayers. Due to the different kinds of chalcogen atoms on both sides of the ZrSSe monolayer, one can find that two different stacking types of ZnO/SZrSe and ZnO/SeZrS vdWHs are formed. We investigate the structural, electronic and optical properties of such vdWHs, as well as the effects of the stacking layers, electric field and interlayer coupling. We find that all of the stacking configurations of ZnO/ZrSSe vdWHs posses type-II band alignment, which can separate the photogenerated electrons–holes. Furthermore, the electric field and vertical strain controlled by adjusting interlayer distances can adjust the band alignment and band gap values of ZnO/ZrSSe vdWHs for both stacking models, making them suitable for high-efficiency electronic and optoelectronic devices.

## 2 Computational methods

In the present work, all the calculations of geometric optimization and electronic properties are obtained from first-principles based on density functional theory (DFT) within the open-source Quantum Espresso package.^50,51^ The exchange–correlation energy is described by the generalized gradient approximation (GGA), which was obtained from the Perdew–Burke–Ernzerhof (PBE) parameterization^52^ using the ultrasoft pseudopotentials (USPP). The cut-off energy is selected to be 500 eV for describing the wave functions in all calculations. Additionally, for the sake of avoiding interactions between two adjacent monolayers, a large vacuum thickness of 35 Å is induced along the *z*-direction of the heterostructures. Furthermore, to overcome the limitation of the standard DFT method for describing the weak vdW interactions, existing in the layered vdWHs, we used the dispersion corrected DFT-D3 method, which was proposed by Grimme *et al.*.^53^ The Brillouin zone sampling of 9 × 9 × 1 was adopted for Monkhorst–Pack *k*-point grids. The energy and force criterion is set to be 10^6^ eV and 0.001 eV Å^−1^, respectively. Spin–orbit coupling (SOC) effects are significant in Janus monolayers. For the ZrSSe monolayer, we previously demonstrated that the SOC effects tend to cause the appearance of band splitting in the valence band of ZrSSe monolayer, leading to its band gap reduction.^38^ However, it should be noted that although the SOC effects give rise to a splitting of the bands and reduce the band gap value of the ZnSSe monolayer, it hardly changes the shape of the bands.^54^ Therefore, the SOC effects are not considered in the following calculations because they have little significant change in the band dispersion of the ZnO/ZrSSe heterostructure.

For the G_0_W_0_ calculation, we use the 6 × 6 × 1 *Γ*-centered *k*-point sampling. The cutoff energy for the plane waves and the response function is set to 410 eV and 200 eV, respectively. The dielectric functions of the heterostructure are calculated using the 8 highest valence bands and the 8 lowest conduction bands, which are set to be a basis for the excitonic states.

## 3 Results and discussion

**Fig. 1:**
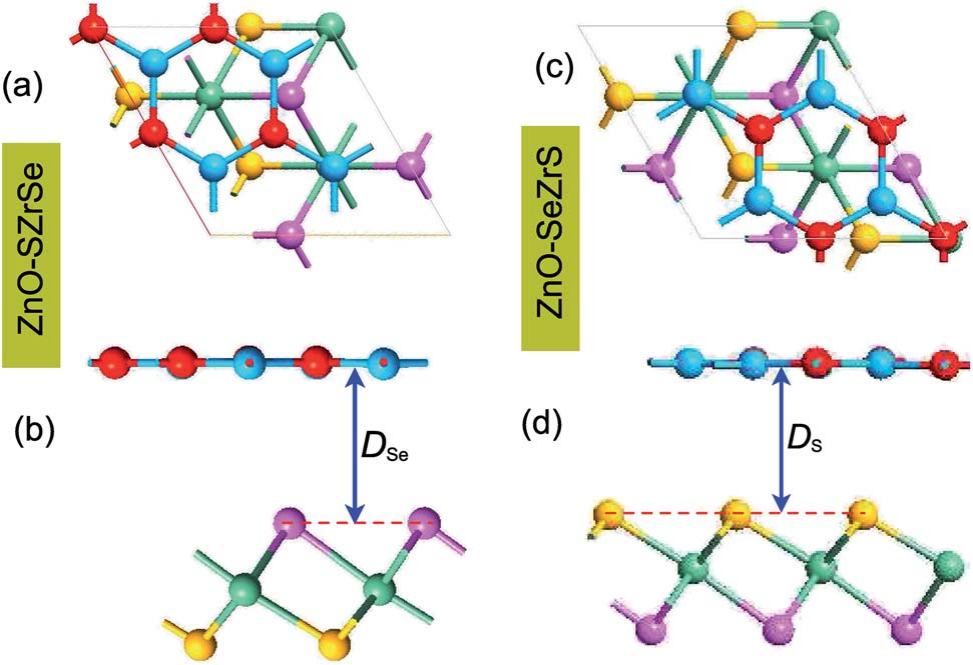
Atomic structure of the ZnO/SZrSe and ZnO/SeZrS heterostructures in different views (a and c) top view and (b and d) side view. The blue and red balls represent the Zn and O atoms, respectively, while purple, green and yellow balls represent the Se, Zr and S atoms, respectively.

The atomic structures of both ZnO and Janus ZrSSe monolayers are fully optimized to obtain the lattice parameters, which are calculated to be 3.25 Å and 3.74 Å, respectively. These values are in good agreement with previous reports,^37,55^ confirming the reliability of our computational methods. To construct the ZnO/ZrSSe vdWH we use a large supercell, which contains (2 × 2) unit cells of monolayer ZnO and 
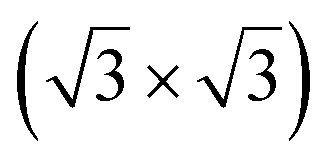
 unit cells of monolayer ZrSSe. The lattice parameters of ZnO and ZrSSe supercells are 6.50 Å and 6.48 Å, respectively, resulting in a tiny lattice mismatch of 0.15%, which is negligible. The atomic structure of two representative kinds of ZnO/ZrSSe vdWHs, *i.e.*, ZnO/SZrSe and ZnO/SeZrS vdWHs, are depicted in Fig. 1. In the ZnO/SZrSe vdWH, the ZnO layer is placed directly on top of the sulfur layer, while in the ZnO/SeZrS vdWH, the ZnO layer is located on top of the selenium layer. After the optimization process, we obtain the interlayer distance *D*_Se_ and *D*_S_, which are calculated to be 3.16 Å and 3.12 Å, respectively. One can find that such values of the interlayer distances are the same as those in other 2D vdWHs, such as graphene/PtSSe,^56^ and GeC/MoSSe.^54^

**Fig. 2:**
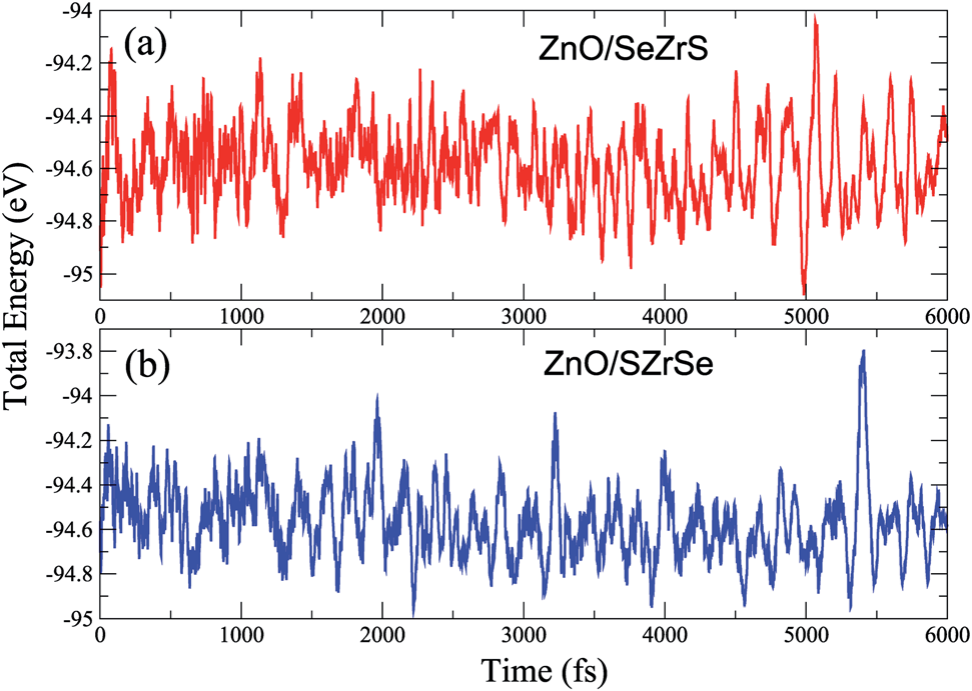
The *ab initio* molecular dynamics calculations of (a) ZnO/SeZrS and (b) ZnO/SZrSe vdWHs at room temperature (300 K).

Furthermore, the binding energy is also examined to confirm the stability of the systems. The binding energy can be calculated as follows:1
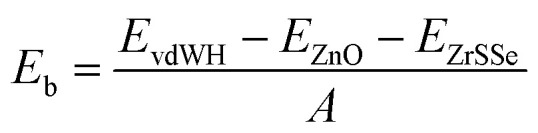
Here, the total energies of vdWH, isolated ZnO and ZrSSe monolayers are denoted by *E*_vdWH_, *E*_ZnO_ and *E*_ZrSSe_, respectively. *A* represents the in-plane surface area of such vdWHs. Our calculated binding energy of ZnO/SZrSe and ZnO/SeZrS vdWHs are −13.25 meV Å^−2^ and −15.29 meV Å^−2^, respectively. The negative values of the binding energies demonstrate that both the ZnO/SZrSe and ZnO/SeZrS vdWHs are energetically stable. Furthermore, we also perform the *ab initio* molecular dynamics calculation for both models of ZnO/ZrSSe vdWHs at room temperature. These results are displayed in Fig. 2. One can see that the variations in the total energies of ZnO/SeZrS and ZnO/SZrSe vdWHs are small with no structural distortion after 6 ps, confirming their excellent thermal stability at room temperature of such vdWHs.

**Fig. 3:**
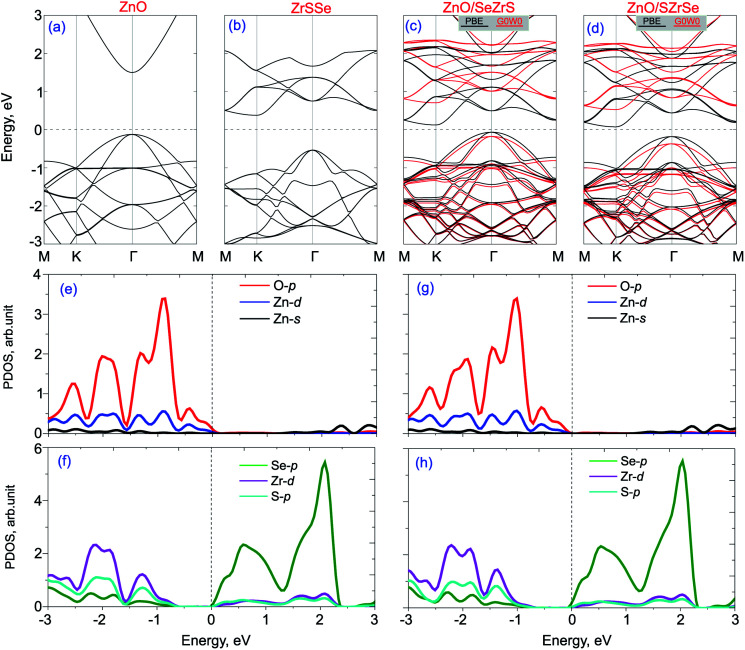
Band structures of isolated (a) ZnO, (b) ZrSSe monolayers and the corresponding (c) ZnO/SeZrS and (d) ZnO/SZrSe vdWHs. (e–h) are the projected density of states of the ZnO and ZrSSe parts in their corresponding vdWHs – ZnO/SeZrS (e–f) and ZnO/SZrSe (g–h).

The band structures of isolated ZnO and Janus ZrSSe monolayers are calculated and plotted in Fig. 3(a) and (b) for comparison. One can see that the monolayer ZnO exhibits a semiconductor direct band gap, where both the conduction band minimum (CBM) and valence band maximum (VBM) are located at *Γ* point, as depicted in Fig. 3(a). While the monolayer ZrSSe possesses an indirect band gap nature with the CBM at the *K* point and VBM at *Γ* point, as depicted in Fig. 3(b). The PBE and G_0_W_0_ band structures of both ZnO/SeZrS and ZnO/SZrSe vdWHs are depicted in Fig. 3(c) and (d), respectively, along with their projected density of states (PDOS) in Fig. 3(e, f) and (g, h), respectively. One can see that both the ZnO/SeZrS and ZnO/SZrSe vdWHs exhibit the indirect band gap semiconductors with the VBM and CBM at the *Γ* and *K* points, respectively. The calculated band gap values for ZnO/SeZrS and ZnO/SZrSe vdWHs given by the PBE/G_0_W_0_ method are 0.25 eV/0.73 eV and 0.19 eV/0.68 eV, respectively. Furthermore, we observe that the band gap value of such vdWHs are narrowed as compared with those of the constituent monolayers, making them easier to excite as the electrons in the VBM towards the CBM require lower energy when the vdWH is under visible light irradiation. More interestingly, comparing the band structures of isolated ZnO and ZrSSe monolayers and the PDOS of ZnO/SeZrS and ZnO/SZrSe vdWHs, one can see from the PBE and G_0_W_0_ band structures that both the ZnO/SeZrS and ZnO/SZrSe vdWHs posses type-II band alignment at the equilibrium state, confirming the accuracy of our theoretical models and the computational details. Although the PBE method underestimates the band gap of materials, it can predict the corrected trends and physical mechanisms of such heterostructures. The VBM of such vdWHs is contributed to by the ZnO layer, whereas the CBM comes from the ZrSSe layer. The PDOS of the ZnO/ZrSSe vdWHs show that the VBM is mainly contributed to by O-p orbitals, while the CBM mainly results from Se-p orbitals. The type-II band alignment makes the ZnO/ZrSSe vdWHs promising candidates for the use of photovoltaic devices because the photogenerated electrons–holes are separated at the interface.

**Fig. 4:**
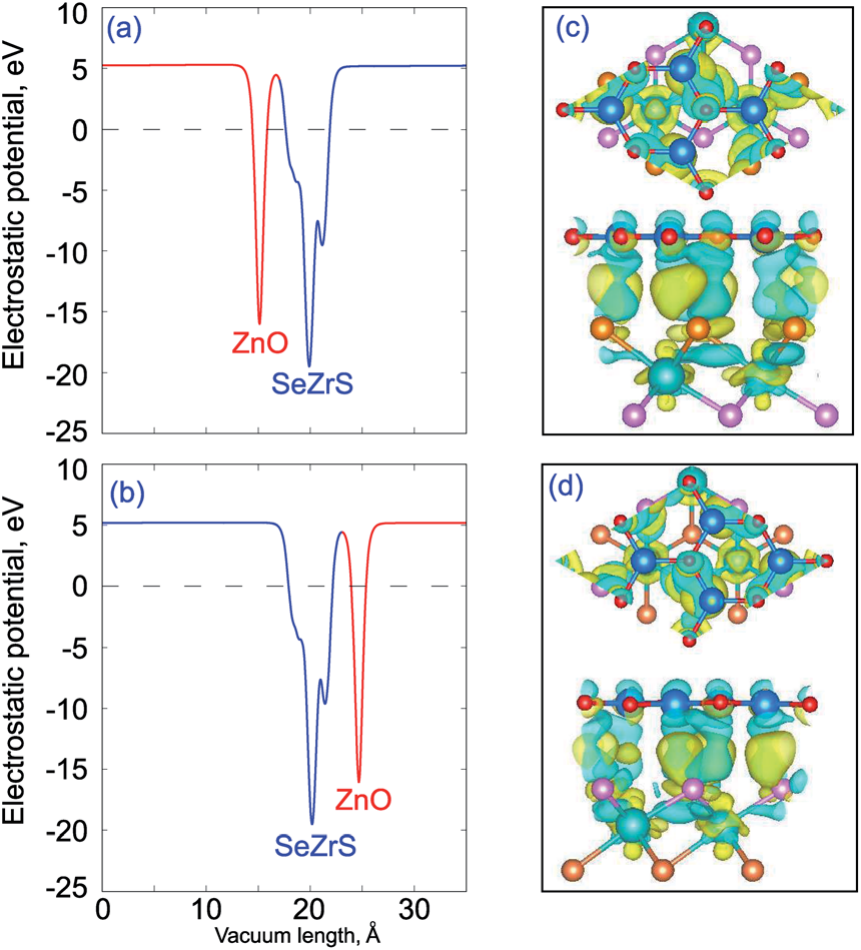
Electrostatic potentials (a and b) and charge density difference (c and d) of the ZnO/SeZrS (a and c) and ZnO/SZrSe (b and d) vdWHs. Yellow and cyan areas represent the electron accumulation and depletion, respectively.

Fig. 4 shows the electrostatic potentials and charge density difference of the ZnO/ZrSSe vdWHs for both stacking patterns. The charge density difference can be obtained by:2
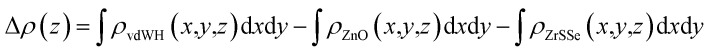
Here, *ρ*_vdWH_(*x*,*y*,*z*)d*x*d*y*, *ρ*_ZnO_(*x*,*y*,*z*)d*x*d*y* and *ρ*_ZrSSe_(*x*,*y*,*z*)d*x*d*y*, are the charge densities of the corresponding vdWH, and the isolated ZnO and ZrSSe monolayers. We can find that the ZrSSe monolayer has a deeper potential than that of the ZnO monolayer for both ZnO/SZrSe and ZnO/SeZrS vdWHs, as depicted in Fig. 4(a) and (b). It indicates that the charge transportation flows from the ZnO layer to the ZrSSe layer. The charge density difference shown in Fig. 4(c) and (d) demonstrates that the charge redistribution mainly occurred at the interface region. By Bader analysis, we find that the ZnO layer donates 0.032*e* and 0.041*e* to the SeZrS and SZrSe layers in the corresponding ZnO/SeZrS and ZnO/SZrSe vdWHs. This means that the charge transfer from the ZnO to Se layer is smaller than that to the S layer. Although the number of transferred electrons from ZnO to the ZrSSe layers is small, a large potential difference between the ZnO and ZrSSe layers creates a built-in electric field, directing from the ZrSSe layer to the ZnO layer. This gives rise to the drift of electrons and holes, eventually reaching dynamic equilibrium with diffusion.

**Fig. 5:**
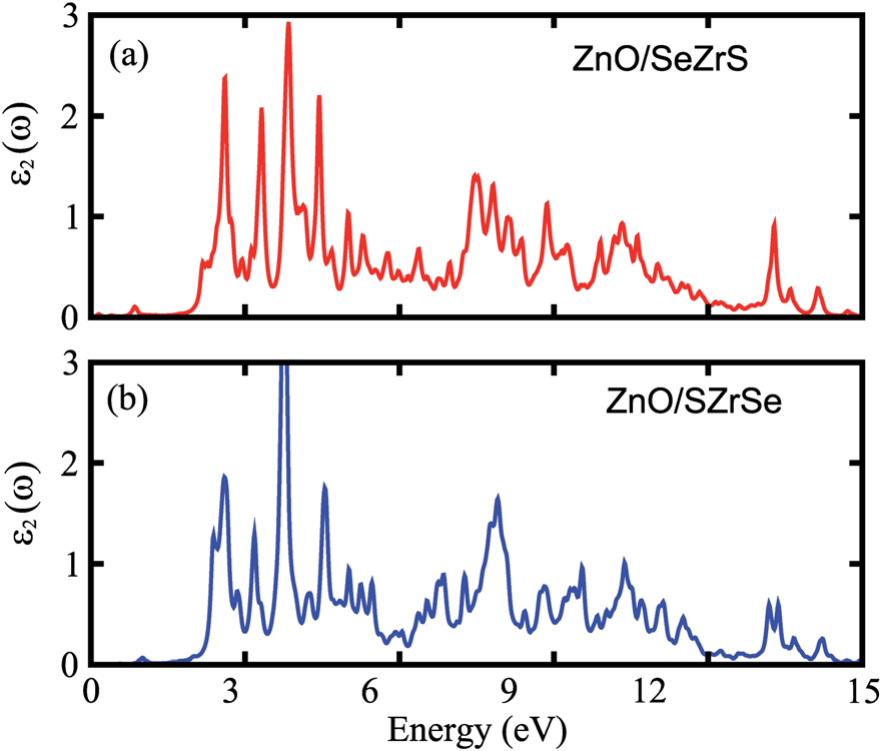
The imaginary part of the dielectric functions of the (a) ZnO/SeZrS and (b) ZnO/SZrSe vdWHs.

We further calculate the optical absorption behavior of the ZrSSe/ZnO vdWHs heterostructures for both models of vdWHs, which are shown in Fig. 5. The optical features are calculated using the Bethe–Salpeter equation (BSE) on top of single-shot G_0_W_0_ calculations as follows:3
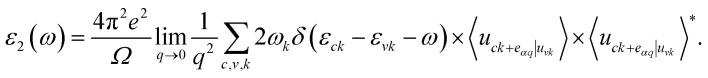
Where, *c* and *v* are the CBM and VBM of the vdWHs, respectively. *u*_*ck*_ represents the cell periodic part of the wavefunctions. One can find that the ZnO/ZrSSe vdWHs have absorption peaks at 539.06 nm and 545.35 nm in the visible light region, and there are several peaks in the ultraviolet region. It can be clearly seen that the ZnO/ZrSSe vdWHs for both models possess high performance absorption in the visible and near-infrared regions, revealing their use for designing efficient photocatalysts.

**Fig. 6:**
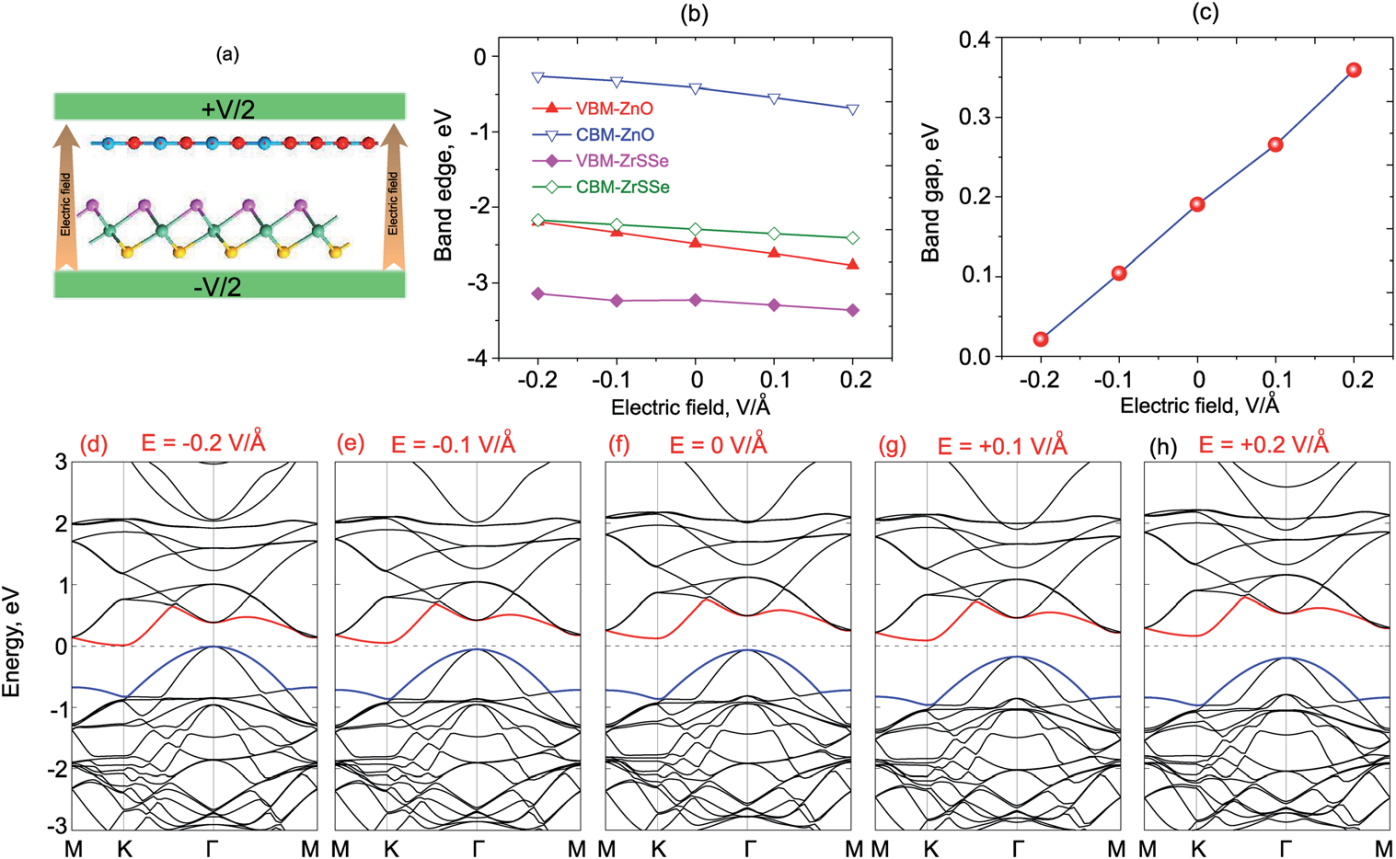
(a) Schematic model of the ZnO/SeZrS vdWH with applied electric field. The variations of the band edge positions (b), and band gap value (c), of ZnO/SeZrS vdWH as a function of electric field. (d–h) The band structures of ZnO/SeZrS vdWH under different electric fields.

**Fig. 7:**
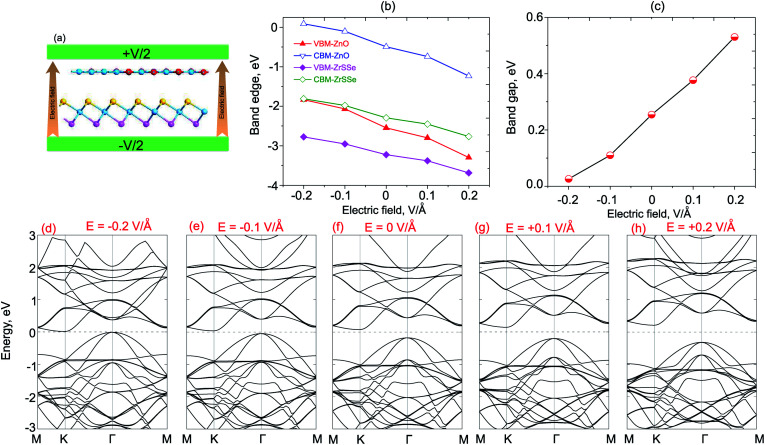
(a) Schematic model of the ZnO/SZrSe vdWH with applied electric field. The variations of the band edge positions (b), and band gap value (c), of ZnO/SZrSe vdWH as a function of electric field. (d–h) The band structures of ZnO/SZrSe vdWH under different electric fields.

What is more, it is interesting that the performance of high-efficiency nanodevices based on 2D vdWH depends strongly on its electronic structure and band alignment, which can also be adjusted by applying electric field or vertical strain by changing the interlayer distances.^55,57,58^ Therefore, we further consider the effects of electric field and vertical strain on the electronic properties and band alignment of both ZnO/SeZrS and ZnO/SZrSe vdWHs. The electric field is applied perpendicularly to the in-plane surface of the heterostructure with the positive direction pointing from ZrSSe to the ZnO layers, as illustrated in Fig. 6(a) and 7(a) for ZnO/SeZrS and ZnO/SZrSe vdWHs. The evolution of the band edge positions relative to the Fermi level (VBM and CBM) under the electric field is depicted in Fig. 6(b) and 7(b) for ZnO/SeZrS and ZnO/SZrSe vdWHs, respectively. We find that the band edge positions of ZnO/SeZrS vdWH depends on the direction of the electric field in two different ways for both cases of ZnO/SeZrS and ZnO/SZrSe vdWHs. The positive electric field tends to decrease the VBM and CBM of both the ZnO and ZrSSe parts of the ZnO/SeZrS vdWH, whereas the negative electric field leads to an increase in the VBM and CBM. The band gap values of both ZnO/SeZrS and ZnO/SZrSe vdWHs almost increase with increasing electric field from −0.2 V Å^−1^ to +0.2 V Å^−1^. With the negative electric field of −0.2 V Å^−1^, the band gap values of both ZnO/SeZrS and ZnO/SZrSe vdWHs are reduced to approximately zero. This indicates that the transition from semiconductor to metal can be achieved when the negative electric field is smaller than −0.2 V Å^−1^. The nature of the changes in the band gap of the ZnO/ZrSSe vdWHs can be explained as follows: the negative external and internal (built-in) electric fields have the same direction, pointing from the ZrSSe to the ZnO layers, thus the total strength of the electric fields is increased, and they cause a decrease in the band gap values of the heterostructures. On the contrary, the positive external electric field is opposite to the internal electric field, resulting in an increase in the band gap values.

To get more detail on the underlying mechanism of the effects of electric field on the electronic properties, we plot the band structures of both ZnO/SeZrS and ZnO/SZrSe vdWHs under different strengths of the positive and negative electric fields, as displayed in Fig. 6(d–g) and 7(d–g), respectively. We can find that the ZnO/SeZrS and ZnO/SZrSe vdWHs have the same changing trends in the band structures under the electric fields. The negative electric field tends to downshift/upshift the CBM/VBM towards the Fermi level. On the contrary, the VBM and CBM of such vdWHs move upwards/downwards away from Fermi level. Thus, the band gap values of such vdWHs increase linearly with increasing electric field from −0.2 V Å^−1^ to +0.2 V Å^−1^. Interestingly, with the positive electric field of −0.2 V Å^−1^, both the VBM and CBM of vdWHs nearly cross the Fermi level, resulting in the transition from semiconductor to metal. Therefore, we can conclude that the electronic properties and band edge positions of the ZnO/ZrSSe vdWHs can be adjusted by applying an electric field and the semiconductor–metal transition can emerge with an applied negative electric field.

**Fig. 8:**
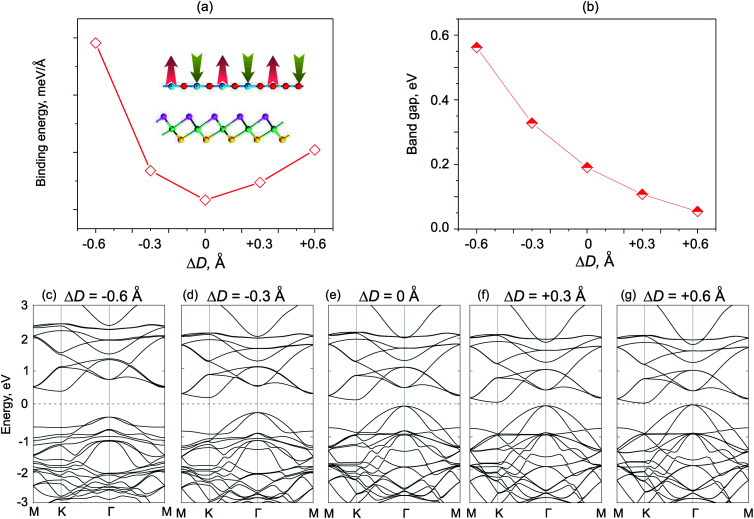
The dependence of the total energies (a), and band gap values (b), of ZnO/SeZrS vdWH under strain. The inset in (a) shows a schematic model of the vertical strains decreasing/increasing interlayer distances. (c–g) The band structures of ZnO/SeZrS vdWH under different vertical strains, Δ*D*.

**Fig. 9:**
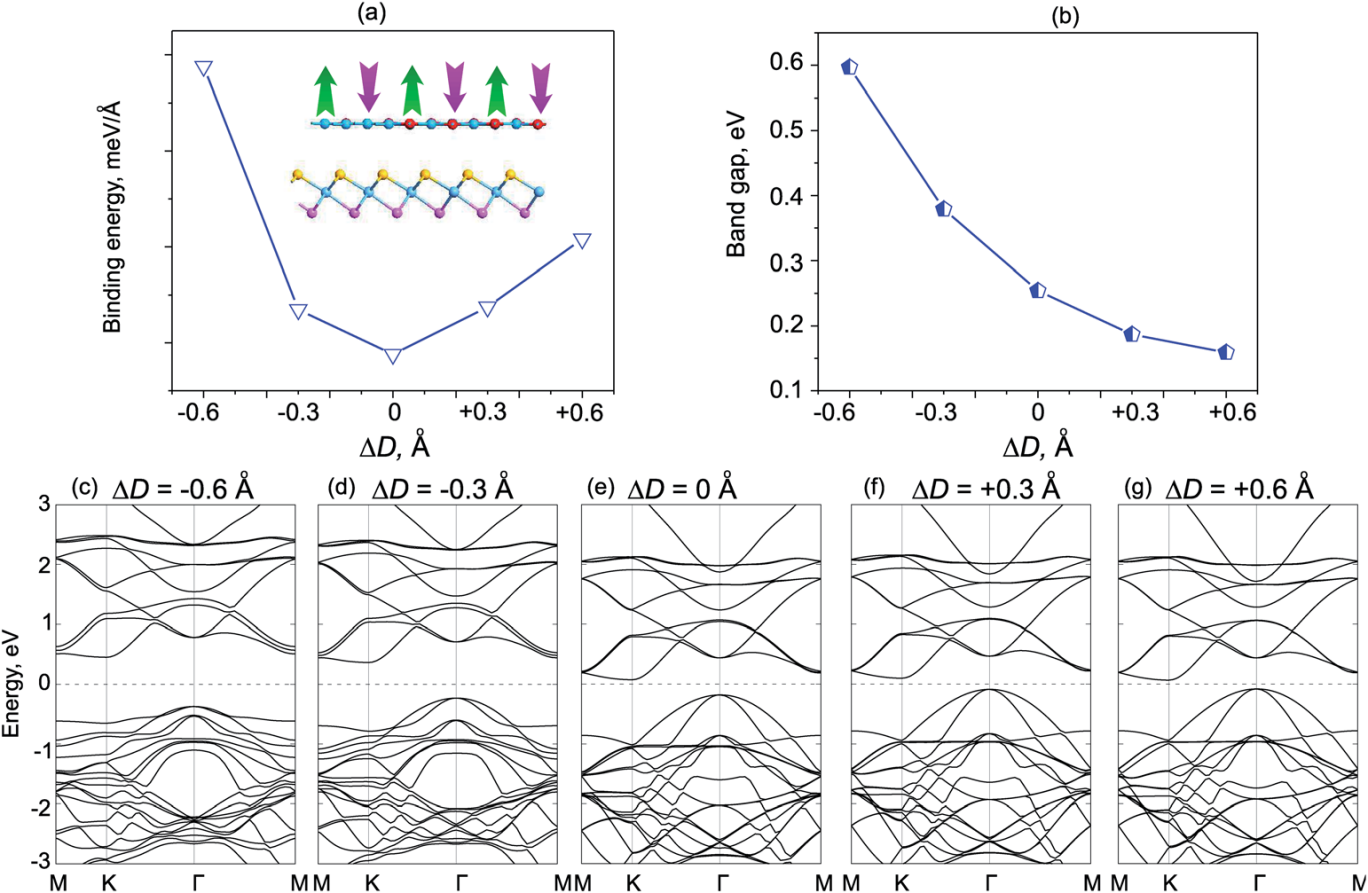
The dependence of the total energies (a), and band gap values (b), of ZnO/SZrSe vdWH under strain. The inset in (a) shows a schematic model of the vertical strains by decreasing/increasing interlayer distances. (c–g) The band structures of ZnO/SZrSe vdWH under different vertical strains, Δ*D*.

We next consider the effects of vertical strain on the electronic properties for both ZnO/SeZrS and ZnO/SZrSe vdWHs, as depicted in Fig. 8 and 9, respectively. The vertical strains are applied perpendicularly to the in-plane surface of the heterostructure by changing the interlayer distances as: Δ*D* = *D* − *D*_0_, where *D* and *D*_0_ are the unstrained and strained interlayer distances, respectively. One can observe that the compressive vertical strain tends to an increase in the band gap values for both stacking models, as illustrated in Fig. 8(b) and 9(b). While, the band gap values of such vdWHs for both models decrease with increasing interlayer distance, *i.e.*, with applied tensile strain. One can see that the band gap values of ZnO/ZrSSe vdWHs for both models decrease to approximately zero under the tensile strain of Δ*D* = +0.6 Å. Our results indicate that the semiconductor to metal transition can be achieved by further increasing the tensile strain up to Δ*D* = +0.70 Å and Δ*D* = +0.72 Å for the ZnO/SeZrS and ZnO/SZrSe vdWHs, respectively. To further understand the changing trends in electronic properties of ZnO/ZrSSe vdWHs for both stacking models, we plot their band structures under different Δ*D*, as illustrated in Fig. 8(c–g) and 9(c–g). We find that under compressive strain, *i.e.*, Δ*D* < 0, both the CBM and VBM of the ZnO/ZrSSe vdWHs move away from Fermi level, leading to an increase in the band gap values. On the contrary, under the tensile strain, both the VBM and CBM of such ZnO/ZrSSe vdWHs for both stacking models shift towards the Fermi level, leading to a decrease in the band gap values. Especially, with Δ*D* = +0.6 Å, as depicted in Fig. 8(g), one can observe that both the VBM and CBM of the ZnO/SeZrS vdWH nearly cross the Fermi level. Thus, we can conclude that the vertical strain, by adjusting interlayer distance, can adjust the band alignment and band gap values of ZnO/ZrSSe vdWHs for both stacking models, making them suitable for multifunctional devices.

## 4 Conclusion

In summary, we have investigated the structural, electronic and optical features of ZnO/ZrSSe vdWHs for different stacking patterns of ZnO/SeZrS and ZnO/SZrSe by employing first-principles calculations. The structural and thermal stabilities of both ZnO/SeZrS and ZnO/SZrSe vdWHs are confirmed by calculating the binding energies using *ab initio* molecular dynamics calculations. Our calculations demonstrated that the ZnO/ZrSSe vdWHs for both models are characterized by weak vdW interactions. Furthermore, the band gap value of such vdWHs are narrowed as compared with those of the constituent monolayers, making them more convenient because the excitation behavior of the electrons from VBM towards CBM requires lower energy when the vdWH is under visible light irradiation. More interestingly, both the ZnO/SeZrS and ZnO/SZrSe vdWHs posses type-II band alignment, making them promising candidates for use in photovoltaic devices because the photogenerated electrons–holes are separated at the interface. The ZnO/ZrSSe vdWHs for both models possess high performance absorption in the visible and near-infrared regions, revealing their use in designing efficient photocatalysts. Moreover, we find that the band alignment and band gap values of ZnO/ZrSSe vdWHs for both models are very sensitive to external electric fields and vertical strains. The transition from semiconductor to metal is also achieved under a negative electric field or tensile vertical strain. These findings demonstrate that the ZnO/ZrSSe vdWHs are promising options for optoelectronic and nanoelectronic applications.

## Conflicts of interest

There are no conflicts to declare.

## Supplementary Material
